# Screening of placenta accreta spectrum disorder using maternal serum biomarkers and clinical indicators: a case–control study

**DOI:** 10.1186/s12884-023-05784-2

**Published:** 2023-07-11

**Authors:** Jiayi Zhou, Si Yang, Xingneng Xu, Xiuting Xu, Xuwei Wang, Anqi Ye, Yanhong Chen, Fang He, Bolan Yu

**Affiliations:** 1 Department of Obstetrics and Gynecology, The Third Affiliated Hospital of Guangzhou Medical University, Guangzhou, China; 2 Guangdong Provincial Key Laboratory of Major Obstetric Diseases; Guangdong Provincial Clinical Research Center for Obstetrics and Gynecology; Guangdong-Hong Kong-Macao Greater Bay Area Higher Education Joint Laboratory of Maternal-Fetal Medicine, The Third Affiliated Hospital of Guangzhou Medical University, Guangzhou, China; 3 BioResource Research Center, The Third Affiliated Hospital of Guangzhou Medical University, No.63 Duobao Road, Guangzhou, Guangdong 510150 China

**Keywords:** Screening model, Placenta accreta spectrum, Biomarkers

## Abstract

**Background:**

Placenta accreta spectrum (PAS) disorder is a major cause of postpartum hemorrhage-associated maternal and fetal death, and novel methods for PAS screening are urgently needed for clinical application.

**Methods:**

The purpose of this study was to develop new methods for PAS screening using serum biomarkers and clinical indicators. A total of 95 PAS cases and 137 controls were enrolled in a case–control study as cohort one, and 44 PAS cases and 35 controls in a prospective nested case–control study were enrolled as cohort two. All subjects were pregnant women of Chinese Han population. Biomarkers for PAS from maternal blood samples were screened based on high-throughput immunoassay and were further validated in three phases of cohort one. Screening models for PAS were generated using maternal serum biomarkers and clinical indicators, and were validated in two cohorts. The expression levels of biomarkers were analyzed using histopathological and immunohistochemical (IHC) techniques, and gene expression was examined by QPCR in the human placenta. Binary logistic regression models were built, and the area under the curve (AUC), sensitivity, specificity, and Youden index were calculated. Statistical analyses and model building were performed in SPSS and graphs were generated in GraphPad Prism. The independent-sample* t* test was used to compare numerical data between two groups. For nonparametric variables, a Mann–Whitney *U* test or a *X*^2^ test was used.

**Results:**

The results demonstrated that the serum levels of matrix metalloproteinase-1 (MMP-1), epidermal growth factor (EGF), and vascular endothelial growth factor-A (VEGF-A) were consistently higher, while the level of tissue-type plasminogen activator (tPA) was significantly lower in PAS patients compared with normal term controls and patients with pre-eclampsia (PE) and placenta previa (PP). IHC and QPCR analysis confirmed that the expression of the identified biomarkers significantly changed during the third trimester in human placenta. The generated screening model combining serum biomarkers and clinical indicators detected 87% of PAS cases with AUC of 0.94.

**Conclusions:**

Serum biomarkers can be used for PAS screening with low expense and high clinical performance; therefore, it may help to develop a practicable method for clinical prenatal PAS screening.

**Supplementary Information:**

The online version contains supplementary material available at 10.1186/s12884-023-05784-2.

## Background

Placenta accreta spectrum (PAS) disorder is defined as abnormal trophoblastic adhesion or invasion of the placenta into the myometrium of the uterine wall, and it is a major cause of postpartum hemorrhage-associated maternal and fetal death [1, 2]. A recent systematic review has reported that the prevalence rates of PAS range from 0.01% to 1.1%, with an overall pooled prevalence of 0.17%; there has been a rapid increase in the PAS prevalence in most areas of the world [3]. For instance, in the United States, the incidence of PAS was 0.19% in 2005, which was eight-fold higher than that in the 1970s and five-fold higher than that in the 1980s [4]. In Europe, the incidence of PAS between 2003 and 2010 increased from 0.165% to 0.237% in women with a prior caesarean delivery (CD) [5]. In mainland China, the reported prevalence of PAS ranges from 0.26% to 0.80% [6].

In clinical practice, optimal management of PAS involves a standardized approach with a comprehensive multidisciplinary care in a center of excellence, and delivery at 34 to 36 weeks of gestation is strongly recommended [7]. Because there are no particular symptoms before onset of labor or bleeding in women with PAS, the primary antenatal diagnostic methods in clinical practice include obstetric ultrasonography and magnetic resonance imaging (MRI) [1]. However, due to the heterogeneous nature of PAS and the expertise and expense required for an imaging diagnosis, at least a half to two thirds of PAS cases remain undiagnosed before delivery [8]. The resulting delay in treatment may cause serious adverse pregnancy outcomes (APO) and increased maternal mortality [7].

Currently, there are no clinical serum biomarkers for prenatal PAS screening. Recent studies have reported potential biomarkers, such as maternal serum α-fetoprotein [9], pregnancy-associated plasma protein A [10], pro B-type natriuretic peptide [11], free β-human chorionic gonadotropin mRNA [12], and total placental cell-free mRNA [13]. The placental tumor necrosis factor-related apoptosis-inducing ligand receptor 2 [14] and circulating levels of VEGF, soluble fms-like tyrosine kinase 1 (sFIT-1) [15], median antithrombin III, median plasminogen activator inhibitor 1, soluble Tie2, and soluble VEGF receptor 2 were also found to be associated with PAS [16]. In addition, maternal VEGF levels were shown to inversely correlate with the clinical degree of invasive placenta [17]. However, due to limited sample sizes and variable reliability among different studies, few of these biomarkers have entered further investigation or clinical application [18]. In this study, we used high-throughput immunoassay to screen serum biomarkers, built screening models which were validated in two case–control studies, and the aim of current study was to develop new methods for prenatal PAS screening using serum biomarkers and clinical indicators.

## Methods

### Study design

This study was conducted with the approval (No. 2014[085], approval date August 2014) of the Ethics Committee of the Third Affiliated Hospital of Guangzhou Medical University. The research was carried out according to the Declaration of Helsinki, and informed consent was obtained from all participants. Study subjects were pregnant women of Chinese Han population who delivered at the Provincial Center for Critical Pregnant Women at the Third Affiliated Hospital of Guangzhou Medical University from August 2015 to December 2020. PAS cases were diagnosed based on both intraoperative diagnosis at delivery and postpartum histopathological analysis according to literature [19], and only cases of placenta increta and placenta percreta with both reports were included in this study. Normal term (NOR) controls were healthy pregnant women with a single fetus and without pre-eclampsia (PE), placenta previa (PP), gestational diabetes (GD), or other complications. Simultaneously, we enrolled PE and PP patients with single fetuses and no other complications as disease controls to test the specificity for identified PAS biomarkers. Maternal and fetal clinical parameters, including maternal age, early pregnancy BMI, gravidity, parity, previous CS, systolic blood press, diastolic blood press, blood glucose, blood loss at delivery, gestational week at birth, fetal birth weight, and Apar score were recorded.

There were two cohorts enrolled in this study. Cohort one was a case–control study conducted from August 2015 to December 2017. Cases and controls were selected in woman with scheduled CD delivery, blood sampling was performed at gestational weeks 34 to 39 in the third trimester prior to delivery. After the final PAS diagnosis was confirmed by postpartum histopathological analysis, serum biomarkers were screened based on high-throughput immunoassay. Cohort one was further divided into a screening group, a validation group, and a test group.

**Fig. 1:**
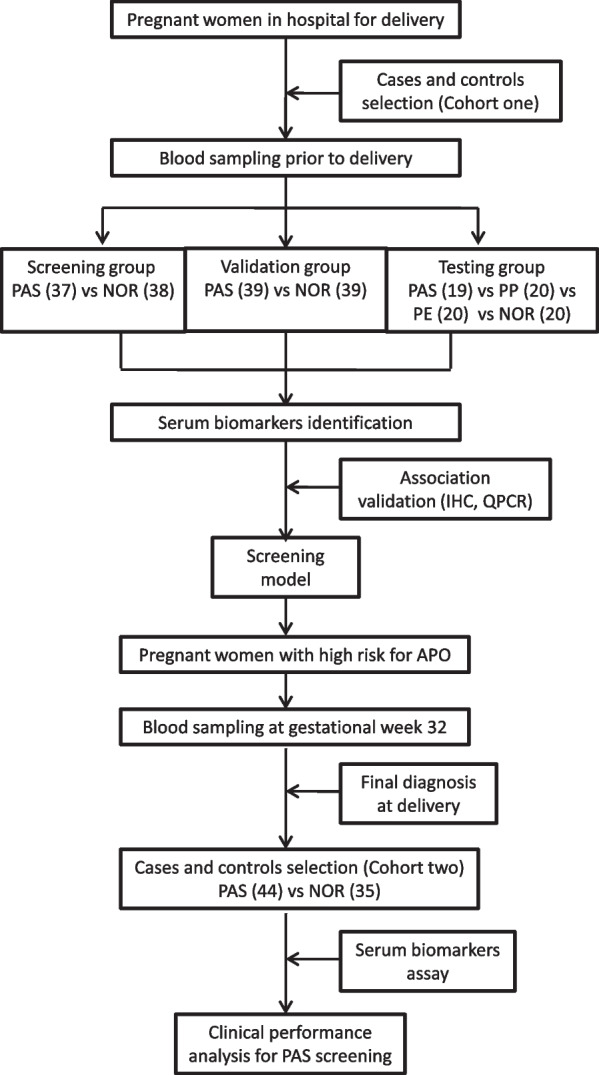
Flowchart of this study. NOR, normal term controls; PAS, pregnant women with placenta accreta spectrum; PE, pregnant women with pre-eclampsia; PP, pregnant women with placenta previa; APO, adverse pregnancy outcomes. The figures between parentheses were the number of enrollment subjects

Cohort two was a prospective nested case–control study of women with a high risk of adverse pregnancy outcomes based on medical records and ultrasound imaging from January 2017 to December 2020. Their blood sampling was collected around gestational week 32. The final diagnosis of all PAS cases was made after delivery, but the results were not available to the technicians who performed the analysis of serum biomarkers. The whole process of this study is demonstrated in Fig. 1.

### Blood sample collection and processing

Venous blood was collected from all of the enrolled subjects using a non-anticoagulant tube. The blood samples were placed at room temperature for 30 min, centrifuged at 2000 g for 15 min to separate serum and blood cells, and stored separately at − 80 °C in the Biobank of the Third Affiliated Hospital of Guangzhou Medical University until subsequent studies. The human placentas from PAS cases and the control group were separated by surgeons after delivery, and the placental tissue from PAS was further divided into parts from the non-implanted area and the implanted area.

### Analysis of serum biomarkers

In this study, we based our strategy for developing serum biomarkers for PAS on three steps. (1) In the study of screening group, we used high-throughput immunoassay kits to screen 103 detectable cytokines and proteins that are known to play major roles in immunity, inflammation, invasion, and angiogenesis in human diseases. (2) We then performed the study of validation group to confirm the findings of serum biomarkers. (3) We also performed the study of testing group to evaluate its specificity for PAS screening in patients with PP and PE, which are pregnancy complications similar to PAS in clinic. In order to ensure the reproducibility of the results, the high-throughput immunoassay kits in these experiments were from different companies; therefore, the ratios of serum levels between cases and controls were the best parameters for comparison.

At the screening phase, we analyzed multiple cytokines using the kits from Bio-Rad Laboratories, Inc. (Hercules, CA, US), which included Pro Human Th17 15-plex Panel, Pro Human Chemokine 40-plex Panel, PRO HU CANCER1, 16-PLEX, 1X96, PRO HU CANCER2, 18-plex, 1X96, BPLX HU AC PHASE COMPLETE 4 + 5, Pro Human Inflammation Panel 1, 37-plex, Pro Hu AP Panel 9-plex, Pro Hu TIMP 4-plex, and Pro TGF-β 3-Plex Panel. At the validation and test phases, we used Luminex Panel custom panel kits (Invitrogen, Carlsbad, CA, US) and a TGF-beta Premixed Magnetic Luminex Performance Assay (R&D Systems, Inc., Minneapolis, Minnesota, US). The samples were analyzed twice to ensure accuracy of the experimental results, and intra- and inter-assay coefficients of variation (CV) values were all under 15%.

### ELISA of serum biomarkers

The levels of cytokines and proteins in human serum were measured using ELISA kits in accordance with the manufacturer’s instructions (Table S1). The absorbance was measured at 450 nm using a microplate reader. Cytokine and protein concentrations of each subject were transformed into values of multiples of the median (MoMs) for comparison.

### RT-QPCR of gene expression

Total RNA in human placenta was extracted using RNeasy Plus Universal Mini Kit (Qiagen, Germany). About one μg RNA was used for cDNA synthesis using PrimeScript™ RT reagent Kit with gDNA Eraser (Takara, Japan), followed by real-time quantification using GoTaq qPCR Master Mix (Promega, USA) on QuantStudio™ 6 Flex System real-time PCR machine (Applied Biosystems, Germany). The primers for detected gene are listed in Table S2. The RT-QPCR data were shown with value of relative expression is 2^−ΔΔCt^ and normalized with the housekeeping gene *GAPDH*.

### Histopathological and immunohistochemical (IHC) analysis

Histopathological and IHC experiments were conducted on placentas using primary antibodies of mouse monoclonal anti-VEGF (1:50; Sigma, USA), rabbit anti-tPA (1:50; Sigma, USA), rabbit anti-MMP-1 (1:50; Sigma, USA), mouse monoclonal anti-EGF (1:500; Sigma, USA). We arranged the formalin-fixed, paraffin-embedded placentas in blocks of tissue microarray using a tissue arrayer instrument (Mitogen Ltd, Harpenden, UK). Stained slides were examined using an inversion fluorescence microscope and images for analysis were captured by ImageJ software (NIH, USA). The results were graded on a semiquantitative scale: 0 (absence of staining/no color), 1 (weak staining/pale brown color), 2 (distinct staining/dark brown color), 3 (strong staining/brownish-black color). Representative scores were taken from the fetal surface to the maternal surface in each original block (controls and PAS cases) and from the invasion area (PAS cases), and the results were analyzed using X^2^ test among all groups.

### Statistical analysis

We performed all statistical analyses in SPSS software version 20.0 (SPSS, Inc., Chicago, Illinois, US) and generated graphs in GraphPad Prism software version 9.0 (GraphPad Software, San Diego, California, US). For normally distributed variables, results are given as mean ± standard deviation (SD). The independent-sample* t* test was used to compare numerical data between two groups. For nonparametric variables, a Mann–Whitney *U* test or a *X*^2^ test was used for comparison of the level of the target genes expression between the groups. *P* < 0.05 (two-sided probability) was interpreted as statistically significant.

We established a binary logistic regression model to predict the probability of PAS using SPSS. The models’ diagnostic accuracy was assessed by the area under the curve (AUC) with a 95% confidence interval (CI); the optimal cutoff value was determined by maximizing the sum of sensitivity and specificity and minimizing overall error according to the following formula: (square root of the sum[1 − sensitivity]^2^ + [1 − specificity]^2^). Sensitivity, specificity, AUC, positive predictive value (PPV), negative predictive value (NPV), positive likelihood ratio (PLR), negative likelihood ratio (NLR), odds ratio (OR), and Youden index were calculated to evaluate the clinical performance of different diagnostic models.

## Results

### Comparison of clinical parameters

According to final diagnosis, a total of 95 PAS cases, 97 normal term controls, 20 PP cases and 20 PE cases were enrolled in the cohort one, and there were 44 PAS cases and 35 NOR controls enrolled in the cohort two.

The clinical data showed that gravidity, parity, and previous CD were significantly higher in the PAS groups than in the control groups in both cohorts (Tables 1 and 2; all *P* < 0.01). The blood loss at delivery was significantly higher in the PAS groups than in any others groups in both cohorts (Tables 1 and 2; all *P* < 0.001). Factors related to pregnancy outcomes, such as gestational week at birth, and birth weight were significantly lower in the PAS groups than in the NOR groups (Tables 1 and 2; all *P* < 0.001). Gestational week at birth and birth weight were both significantly lower in the PP and PE than in the NOR groups; in contrast, the rates of gravidity, parity, and CD were similar among the three groups, except that the CD rate was higher in the PP group than in normal controls (Table 1).

**Table 1:** Demographic and clinical features of study subjects in the cohort one

	Screening group		Validation group		Testing group			
Group	NOR	PAS	NOR	PAS	NOR	PAS	PP	PE
Number	38	37	39	39	20	19	20	20
Gestational week at blood sampling (wks)	38.90 ± 2.72	34.78 ± 2.34	39.07 ± 2.37	34.61 ± 1.30	39.69 ± 1.01	33.71 ± 4.18	34.46 ± 3.32	36.34 ± 3.02
Maternal age (yrs)	29.37 ± 4.83	32.32 ± 4.56	29.62 ± 4.37	32.62 ± 5.20	29.90 ± 4.24	32.32 ± 5.64	33.15 ± 4.07	31.50 ± 3.59
Early pregnancy BMI	20.27 ± 2.44	22.17 ± 3.77	22.20 ± 2.65	21.28 ± 2.59	20.14 ± 2.59	20.50 ± 2.83	19.78 ± 1.51	21.71 ± 3.24
Gravidity (n) ^a^	2.11 ± 1.25	3.51 ± 1..07**	2.72 ± 1.47	4.00 ± 1.43**	2.75 ± 1.25	3.53 ± 1.47*	2.75 ± 1.33	2.50 ± 1.32
Parity (n) ^a^	0.50 ± 0.60	1.30 ± 0.62**	0.87 ± 0.80	1.23 ± 0.48*	0.85 ± 0.81	1.21 ± 0.54*	0.95 ± 0.59	0.65 ± 0.67
Previous CS (n) ^a^	0.26 ± 0.45	1.22 ± 0.67**	0.38 ± 0.54	1.18 ± 0.45**	0.35 ± 0.59	1.06 ± 0.60*	0.70 ± 0.47^#^	0.40 ± 0.50
Systolic blood press (mmHg) ^b^	114 ± 9	113 ± 8	115 ± 10	112 ± 11	114 ± 8	110 ± 10	114 ± 11	157 ± 29**
Diastolic blood press (mmHg) ^b^	74 ± 8	70 ± 7	72 ± 8	70 ± 9	72 ± 7	71 ± 8	69 ± 6	100 ± 24**
Blood glucose (mmol/L)	5.14 ± 1.31	5.01 ± 1.04	5.13 ± 1.13	5.16 ± 1.19	5.35 ± 1.10	4.55 ± 0.88	4.66 ± 1.15	4.97 ± 1.27
Blood loss at delivery (mL)	243 ± 99	9542 ± 14,990**	265 ± 119	3074 ± 3677**	280 ± 92	7366 ± 11,926**	968 ± 1140	319 ± 151
Gestational week at birth (wks) ^b^	39.51 ± 0.85	35.75 ± 1.98**	39.78 ± 1.00	35.27 ± 3.90**	40.04 ± 1.00	35.68 ± 2.53**	35.85 ± 2.28**	36.48 ± 2.62**
Birth weight (g) ^b^	3242 ± 372	2753 ± 504**	3377 ± 341	2534 ± 382**	3419 ± 498	2517 ± 558**	2575 ± 508**	2476 ± 974**
Apar score (10 min)	10.00 ± 0.00	9.97 ± 0.16	10.00 ± 0.00	9.72 ± 1.61	10.00 ± 0.00	10.00 ± 0.00	9.95 ± 0.22	9.65 ± 1.18

Data are present as Mean ± S.D. *PAS* Pregnant women with placenta accreta spectrum disorders, *NOR* Normal term controls, *PE* Pregnant women with preeclampsia, *PP* Pregnant women with placenta previa

^a^: compared by nonparametric M-W* U* test. ^b^: compared by unpaired *t*-test

^#^: *P* < 0.05; ^*^: *P* < 0.01; ^**^: *P*<0.001, compared to NOR group

**Table 2:** Demographic and clinical features of study subjects in the Cohort two

Group	NOR	PAS
Number	35	44
Gestational week at blood sampling (wks)	33.34 ± 1.01	32.92 ± 1.11
Maternal age (yrs)	32.37 ± 4.79	33.20 ± 4.28
Early pregnancy BMI	20.63 ± 2.42	21.50 ± 2.69
Gravidity (n) ^a^	2.12 ± 1.02	3.89 ± 1.60**
Parity (n) ^a^	0.49 ± 0.55	1.20 ± 0.73**
Previous CS (n) ^a^	0.06 ± 0.24	0.91 ± 0.60**
Systolic blood press (mmHg) ^b^	120 ± 9	121 ± 12
Diastolic blood press (mmHg) ^b^	76 ± 7	74 ± 8
Blood glucose (mmol/L)	4.86 ± 1.24	5.44 ± 1.40
Blood loss at delivery (mL)	300 ± 230	1493 ± 1402**
Gestational week at birth (wks) ^b^	39.23 ± 0.85	34.03 ± 0.77**
Birth weight (g) ^b^	3179 ± 317	2282 ± 397**
Apar score (10 min)	9.85 ± 0.70	9.88 ± 0.31

Data are present as Mean ± S.D. PAS, pregnant women with placenta accreta spectrum disorders. NOR, normal term controls

^a^: compared by nonparametric M-W* U* test. ^b^: compared by unpaired *t*-test

^#^: *P* < 0.05; ^*^: *P* < 0.01; ^**^: *P* < 0.001, compared to NOR group

### Findings in cohort one and cohort two

In the screening group of Cohort one, we measured 103 candidate cytokines and found that 34 serum biomarkers were significantly different in the PAS group compared with the NOR group (Table S3; all *P* < 0.05). In the validation group, there were seven biomarkers different in the PAS group compared with the NOR group, even when using detection assay kits from different companies (Table S4; all *P* < 0.05). Of these, the levels of CD30, MMP-1, MMP-8, MMP-9, VEGF-A, and EGF significantly increased, while those of tPA significantly decreased (Table S4; all *P* < 0.05).

In the testing group, PE and PP patients were also enrolled because both of these conditions may lead to the occurrence of antenatal hemorrhage or placental abnormalities appearing on ultrasound in clinical practice. We found that EGF, VEGF-A, and MMP-1 were significantly higher in PAS patients than in PP patients, with respective ratios of 1.70, 8.61, and 3.35 (Table S5; all *P* < 0.05). These biomarkers were also higher and tPA was lower in PAS than in PE patients, with ratios of 2.82, 4.52, 2.56, and 0.42 (Table S5; *P* < 0.05 or *P* < 0.1).

**Fig. 2:**
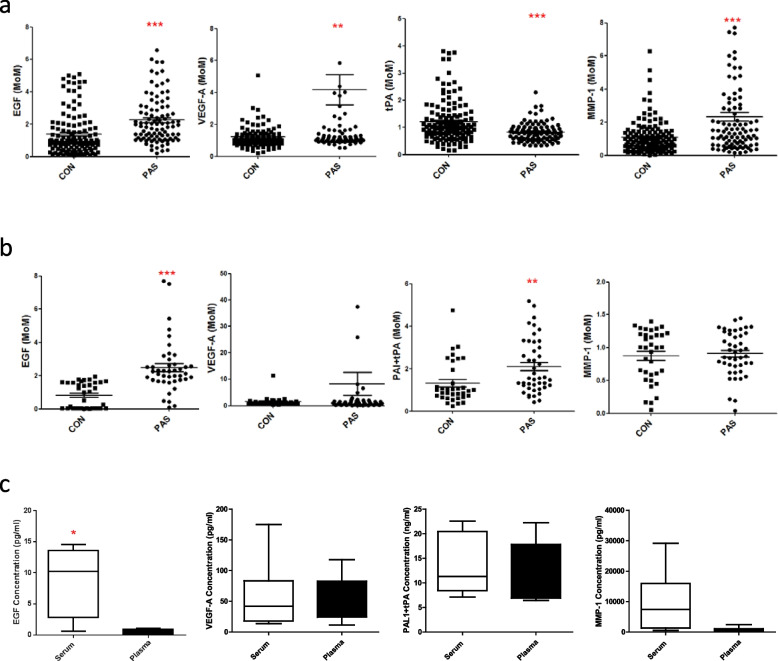
Expression levels of serum biomarkers in different groups. **a** MoMs of EGF, VEGF-A, tPA, and MMP1 in the serum from PAS and CON groups in Cohort one; (**b**) MoMs of EGF, VEGF-A, PAI-tPA, and MMP1 in the serum from PAS and CON groups in Cohort two; (**c**) comparison of the detected levels of EGF, VEGF-A, PAI-tPA, and MMP1 between serum and plasma. MoM: multiples of the median; CON: group of NOR, PE, and PP cases; PAS: PAS cases. *: *P* < 0.01. **: *P* < 0.001, compared with CON group

In Fig. 2, it is demonstrated that the PAS group in cohort one showed significantly higher EGF, VEGF-A, and MMP-1, and significantly lower tPA (Fig. 2a, all* P* < 0.01). In the cohort two, the results showed that EGF and PAI1-tPA (inactivated tPA) were significantly higher in PAS patients than in controls, with respective ratios of 3.03 and 1.59 (Fig. 2b, all* P* < 0.01). VEGF-A showed a strong tendency to higher values in PAS but it did not reach the statistical significance (Fig. 2b). However, there was no alteration in MMP-1 levels (Fig. 2b), probably due to the limited number of cases and different assay kits in cohort two. In addition, the levels of MMP-1, EGF, PAI1-tPA, and VEGF-A in serum and plasma were also compared; there were no significant differences in the results between serum and plasma (Fig. 2c).

### Clinical performance of screening models in different cohorts

**Fig. 3:**
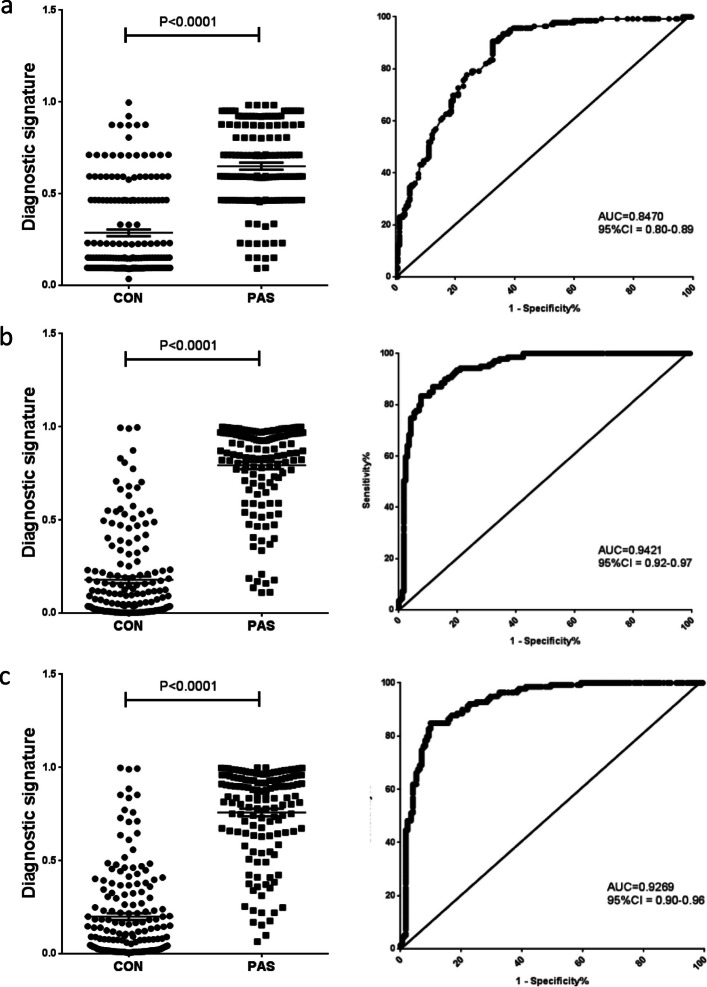
Diagnostic signature of each sample and ROC curve of three screening models in all subjects. (**a**) model M1; (**b**) model M2; (**c**) model M3; ROC: Receiver Operating Characteristic; AUC: the area under the curve; CON: group of NOR, PE, and PP cases; PAS: PAS cases. *: *P* < 0.01. **: *P* < 0.001, compared with CON group

Binary logistic regression models for PAS screening were calculated using different combinations of clinical indicators and serum biomarkers. For calculation, the following factors were applied: the factor X1 was the gestational week of blood sampling, and X2 was the maternal age; factors X3, X4, and X5 were the numbers of previous gravidity, parity, and CD; factors X6, X7, X8, and X9 were the MoM values of serum EGF, VEGF-A, tPA (or PAI1-tPA), and MMP-1. In the transformed model, factor [X2] was the level of maternal age, which was 0 (< 35 years old) or 1 (≧35 years old); factors [X6], [X7], [X8], and [X9] were the levels of MoM values, which were 0 (negative, MoM values < cutoff value) or 1 (positive, MoM values ≧ cutoff value). Factor [X6X8] was the combined level of [X6] and [X8], which was either 0 (negative, one or two MoM values < cutoff value) or 1 (positive, both MoM values ≧ cutoff value). The "diagnostic signature" was calculated using different models in studied subjects to indicate the risk for PAS. Model M1 used four clinical indicators only; model M2 used all clinical indicators and serum biomarkers; and model M3 used normalized parameters. The equations of model M1, M2, and M3 were described in Table 3. The data demonstrated that the diagnostic signature of PAS cases were significantly higher than that of controls in all three models (Fig. 3, all *P* < 0.0001).

**Table 3:** Performance parameters of three models for PAS screening

Model	AUC (95%CI)	Sensitivity (%)	Specificity (%)	PPV (%)	NPV (%)	PLR	NLR	OR	Youden Index
M1^a^	0.847	77.70	76.16	72.48	80.86	3.2595	0.2928	11.13	0.5386
M2^a^	0.9421	87.05	88.37	85.82	89.41	7.4863	0.1465	51.09	0.7542
M3^a^	0.9269	84.89	90.12	87.41	88.07	8.5891	0.1676	51.23	0.7501

*AUC* Area under curve, *PPV* Positive prediction value, *NPV* Negative prediction value, *PLR* Positive likelihood ratio, *NLR* Negative likelihood ratio, *OR* Odd ratio

^a^: Binary logistic regression models were calculated using combinations of clinical indicators and serum biomarkers. Factors applied were described in the results section. Model M1 used four clinical indicators only; model M2 used all clinical indicators and serum biomarkers; and model M3 used normalized parameters. Equations of screening models M1, M2, and M3 were established as following:

M1 = EXP(-2.796 + 0.001*X2 + 0.520*X3-0.542*X4 + 2.122*X5)/{1 + EXP(-2.796 + 0.001*X2 + 0.520*X3 -0.542*X4 + 2.122*X5)}

M2 = EXP(11.151–0.422*X1-0.046*X2 + 0.515*X3-0.904*X4 + 2.331*X5 + 2.050*X6 + 0.204*X7 + 1.068*X8

+ 1.027*X9)/{1 + EXP(11.151–0.422*X1-0.046*X2 + 0.515*X3-0.904*X4 + 2.331*X5 + 2.050*X6 + 0.204*X7

+ 1.068*X8 + 1.027*X9)}

M3 = EXP(10.322–0.383*X1-0.025*[X2] + 0.499*X3-0.773*X4 + 2.216*X5 + 1.849*[X6X8])/{1 + EXP(10.322 -0.383*X1-0.025*[X2] + 0.499*X3-0.773*X4 + 2.216*X5 + 1.849*[X6X8])}

To identify the best model for prenatal screening of PAS, we used different models in all subjects in cohort one and cohort two. The diagnostic performance analysis demonstrated that the combined models could efficiently screen PAS (Table 3; Fig. 3): the AUC was 0.847 if using model M1, but the AUC increased to 0.9421 and 0.9269 if models M2 and M3 were used, respectively. The model M2 had the highest AUC, sensitivity, and Youden Index in all groups (Table 3; Fig. 3). For the model M3, the sensitivity and specificity in all subjects were 84.89% and 90.12%, respectively (Table 3).

### Comparison of different methods for PAS screening

The clinical gold standard for PAS diagnosis is intraoperative observation or postnatal pathological analysis [19]. Primary antenatal diagnostic methods include obstetric ultrasonography and MRI, and antepartum hemorrhage reported by patients is considered a warning sign of PAS. Compared with the sensitivity of antenatal hemorrhage and obstetric ultrasonography, the screening method developed in this study showed great potential for clinical application (Table 4). The data demonstrated that antepartum hemorrhage was a strong sign of PAS, as 65% of PAS patients were positive. About 78% of these patients could be detected by obstetric ultrasonography. Using the models developed in this study, M1 screened 78%, M2 screened 87%, and M3 screened 85% of all PAS cases (Table 4).

**Table 4:** Comparison of different methods for PAS screening in studied subjects

Methods	Positive (%)	Expertise	Expense
Model M1	108(78%)	I	I
Model M2	121(87%)	I	II
Model M3	118(85%)	I	II
Antenatal hemorrhage	90(65%)	I	I
Ultrasonography	109(78%)	III	III

*N* = 139. Data are present as number (percentage) of positive results for PAS screening in studied subjects. All PAS cases were finally confirmed by both intraoperative diagnosis and postpartum histopathological analysis

Expertise: I, No or minimum expertise required; III, some expertise required; III, special expertise required

Expense: I, No or minimum expense required; III, medium expense required; III, high expense required

In addition, the method developed in this study for PAS screening only required minimal expertise and relatively low expense; as in our hospital, the expense for the placental ultrasonic examination is around 60 dollars, but the price of current study is estimated to be 20 dollars (similar to the price of serum aneuploidy screening). Therefore, current method had both low expense and high capability for high-throughput screening, which makes it especially suitable for screening in a large population or women in low-income area.

### Histopathological and QPCR validation

**Fig. 4:**
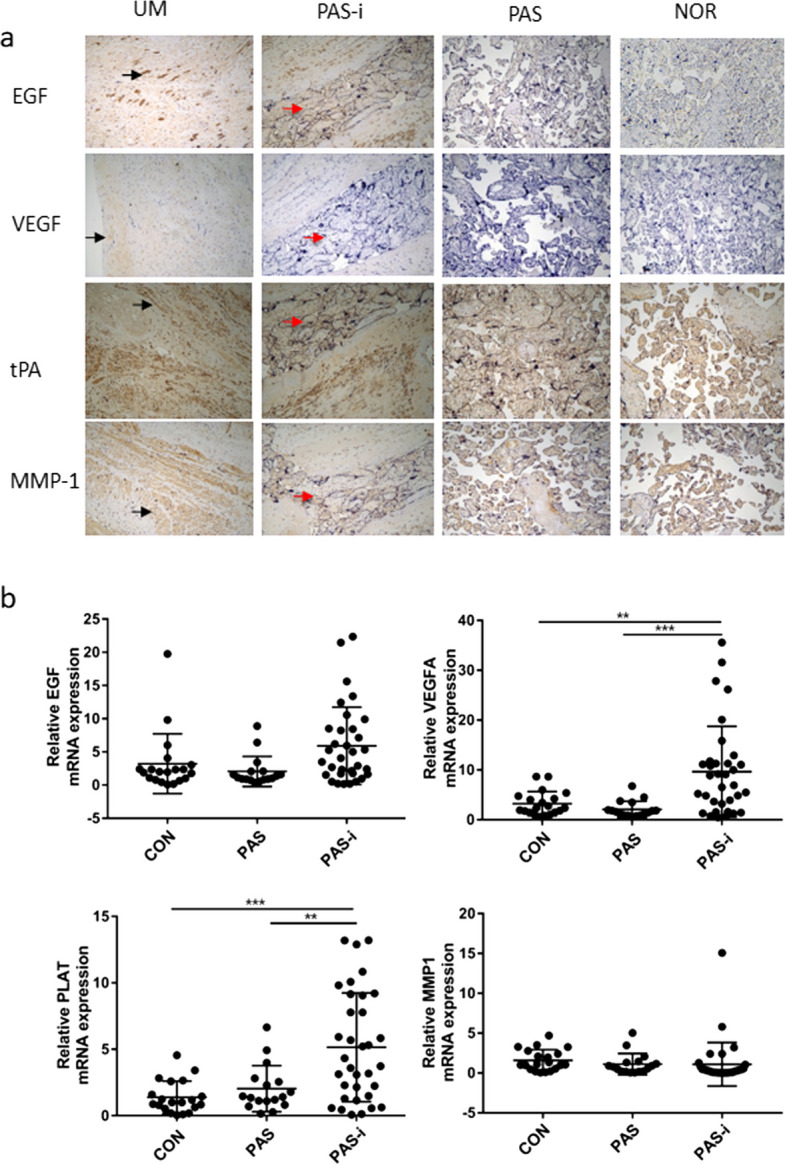
Validation of the altered biomarkers in human placenta. **a** IHC analysis of EGF, VEGF, tPA, and MMP1 in placenta from PAS cases and controls (50 × magnification; black arrows indicate uterus muscle; red arrows indicate invasive placental trophoblasts) (b) QPCR analysis of *EGF*, *VEGFA*, *PLAT(tPA)*, and *MMP1* in placenta from PAS cases and controls. UM: Uterus muscle; CON: placenta from normal term controls; PAS: non-invasion area of placenta from PAS patients; PAS-i: invasion area of placenta from PAS patients

To validate that the alteration of serum biomarkers was associated with PAS, we examined their expression in the different places of the placenta from PAS patients and NOR patients. The histopathological analysis indicated that placental villi invaded the uterine muscles in PAS cases, while villi and uterine muscles were easily separated in the NOR group (Fig. 4a). We further conducted IHC experiments and semi-quantitative analysis of uterine muscle at the location of villus invasion and adjacent placental villi from PAS patients as well as placental villi from NOR group (Fig. 4b); the results confirmed that all four cytokines were elevated in PAS patients (Table S6). VEGF-A was strongly expressed at the location of villus invasion, while EGF, MMP-1, and tPA were more concentrated in the non-implantation placental area in PAS patients (Table S6; all *P* < 0.001).

## Discussion

Clinical studies on PAS are much less common than those on other pregnancy complications such as PE and GD. In limited PAS studies reported so far, there is a large degree of heterogeneity between the studies because of inconsistencies in diagnostic criteria and lack of histopathological confirmation [3]. In this study, we established screening models for PAS using maternal serum biomarkers and clinical indicators in a case–control study, and further validated these models in an independent group of women with high risk for adverse pregnancy outcomes. The results demonstrated that they could effectively screen PAS in both cohorts, and the changed serum proteins were highly associated with abnormal placentation in PAS.

Our study firstly demonstrated that the combined model was more suitable for PAS screening. As PAS is a complex disease without easily distinguished symptoms, the discovery of a single marker that can diagnose PAS before delivery is highly unlikely [7]. In this study, in order to enhance the efficiency of screening, we calculated the risk score for PAS using models with multiple factors rather than using any single biomarker. The results demonstrated that the model combining serum biomarkers and clinical indicators highly improved specificity and sensitivity (Table 3). For instance, in Cohort one, the percentage of positive screening using clinical indicators was 74.1%, while those of single serum biomarkers were 61.4, 65.9, 60.6, and 64.4%; however, the combination of clinical indicators and serum biomarkers yielded the total percentage of 86.2% (unpublished data). In all subjects, with combined markers in a logistic regression model, the AUC increased from 0.85 to 0.93, suggesting a good sensitivity and specificity for PAS screening (Table 3 and Fig. 3). In addition, the results demonstrated that this non-invasive method had higher positive rates of PAS screening compared with current methods such as prenatal hemorrhage and obstetric ultrasonography, which may help to improve the detection rates of PAS in clinic (Table 4).

Although the etiology of PAS remains largely unknown, this study has proven the critical roles of cell invasion, angiogenesis, and coagulability regulation in the onset and development of PAS. Previous studies have suggested that trophoblastic cells constitutively produce MMPs, and the regulation of MMP activity at the maternal–fetal interface is critical for successful implantation and placentation [20]. As a result, aberrant MMPs expression has been found in the gestational diseases fetal growth restriction (FGR) and PE, and MMPs expression increased in PAS patients compared with those with normal pregnancies [21]. Our studies showed that MMP-1 increased in the placenta of PAS patients (Fig. 2), suggesting that the invasion of placental villi into the uterine wall was unique to PAS patients. In addition, invasive extravillous cytotrophoblasts (EVCTs) can excrete angiogenic factors such as angiogenesis factors, including EGF and VEGF; the maternal decidua, defects of which are a major contributing factor to PAS formation, can also express such factors [22]. Indeed, our studies showed that EGF and VEGF-A were highly increased in PAS placenta (Fig. 2), proving that angiogenesis is important to placental invasion, especially in the uterine muscle at the location of villus invasion area and adjacent placental villi. Moreover, normal pregnancy is a state of hypercoagulability with diminished fibrinolytic activity, which is associated with an increase in plasminogen activator inhibitor type 1 and decrease in tPA [23]. Our data also showed that free tPA decreased in circulating plasma in PAS cases (Fig. 2), which might be linked with hemorrhage throughout pregnancy of PAS.

Our study also suggested that inconsistency largely existed in PAS biomarker discovery. For instance, a proteomic study reported that median antithrombin III, median plasminogen activator inhibitor 1, soluble Tie2, and soluble VEGF receptor 2 were significantly dysregulated in PAS compared with controls [16]. In this study, we did not find exactly the same proteins among the discovered biomarkers, but tPA and VEGF-A (Fig. 2) belong to the same protein family of plasminogen activator inhibitor 1 and VEGF receptor 2. In addition, it was reported that maternal serum VEGF can help in predicting abnormally invasive placenta and hint at the degree of invasion [17]. We found that a similar protein, VEGF-A, significantly increased in serum of PAS women compared with normal pregnancy (Fig. 2). We assumed that genetic backgrounds may have contributed to the observed inconsistence. For instance, East Asian women usually have small stature and the pre-pregnancy BMI ranged from 19 to 22 in our study (Tables 1 and 2), which is lower compared with 23 to 28 in White women [18]. In addition, in a number of previous studies, control groups enrolled pregnant women who had a preterm labor [16, 17], while we used the normal term controls in this study. Although the placentation may be normal in the preterm women, it is likely that they also had altered immunity and were different from normal term controls. Finally, screening time may affect the study results. As the structure of human placenta is established around the third week of gestation [24], the inner third of the myometrium is not fully invaded until at least 24 weeks [25]; therefore, the serum biomarkers may be different during different time stages given that PAS may occur at different time points. To diminish such effects, we added blood sampling weeks as a parameter in our model (Table 3).

For prenatal PAS screening, the ultrasound imaging is particularly important, especially for women with previous cesarean scar and at high risk of altered placentation [26]. However, ultrasound screening for placentation is not routinely performed in low-income area, and ultrasound imaging sometimes have limited detection on posterior placenta and twin pregnancies [27, 28]. The serum biomarkers will be of greatly helpful on these women, as its low price, high operability and capability for high-throughput screening make it easily to apply in a large population or low-income area. In future, these serum biomarkers might be developed to use with ultrasound imaging for PAS screening prenatally in a model similar to that used for aneuploidy screening in clinic. However, as this is a study in a single center in Southern China, selection biases are unavoidable. In addition, its applicability is limited because only Asian women were enrolled with small sample size. In future, large clinical trials of these biomarkers in different medical centers are warranted, and the screening of serum biomarkers in the first trimester, the conjunction of ultrasound markers, clinical markers and serum markers, and the application of biomarker screening in twin-pregnancies might be especially important for future clinical utility.

## Conclusions

Based on two case–control studies, we discovered that the combination of serum cytokines and clinical indicators could be a good model for PAS screening. Compared with the current prenatal PAS screening techniques, this method is convenient and inexpensive, with high sensitivity and specificity. Therefore, our research might help to develop a convenient, fast, and economic method for prenatal PAS screening, which would facilitate clinical PAS management and decrease the rates of maternal mortality.

## Supplementary Information

**Additional file 1: Table S1.** Serum biomarkers measured and the commercial ELISA kits used. **Table S2.** Primers for RT-QPCR. **Table S3.** Alterations in serum levels of cytokines and proteins between cases and controls in the screening group of Cohort one. **Table S4.** Confirmation of selected serum biomarkers for PAS screening in the validation group of Cohort one. **Table S5.** Specificity analysis of selected serum biomarkers for PAS screening the testing group of Cohort one. Table S6. Analysis of protein expression levels on human placenta.

## Data Availability

The datasets used and/or analysed during the current study are available from the corresponding author on reasonable request.

## Abbreviations

APO: Adverse pregnancy outcomes
PAS: Placenta accreta spectrum
PE: Pre-eclampsia
PP: Placenta previa
FGR: Fetal growth restriction
GD: Gestational diabetes
CD: Caesarean delivery
IHC: Immunohistochemical
NOR: Normal term controls
AUC: Area under the curve
MMP-1: Matrix metalloproteinase-1
EGF: Epidermal growth factor
VEGF-A: Vascular endothelial growth factor-A
tPA: Tissue-type plasminogen activator
sFIT-1: Soluble fms-like tyrosine kinase 1
MRI: Magnetic resonance imaging
DBP: Diastolic blood pressure
SBP: Systolic blood pressure
CV: Coefficients of variation
MoMs: Multiples of the median
CI: Confidence interval
PPV: Positive predictive value
NPV: Negative predictive value
PLR: Positive likelihood ratio
NLR: Negative likelihood ratio
OR: Odds ratio
EVCTs: Extravillous cytotrophoblasts
