# Correction: Screening of placenta accreta spectrum disorder using maternal serum biomarkers and clinical indicators: a case–control study

**DOI:** 10.1186/s12884-026-09053-w

**Published:** 2026-05-11

**Authors:** Jiayi Zhou, Si Yang, Xingneng Xu, Xiuting Xu, Xuwei Wang, Anqi Ye, Yanhong Chen, Fang He, Bolan Yu

**Affiliations:** 1 Department of Obstetrics and Gynecology, The Third Affiliated Hospital of Guangzhou Medical University, Guangzhou, China; 2 Guangdong Provincial Key Laboratory of Major Obstetric Diseases, Guangdong Provincial Clinical Research Center for Obstetrics and Gynecology, Guangdong‑Hong Kong‑Macao Greater Bay Area Higher Education Joint Laboratory of Maternal‑Fetal Medicine, The Third Affiliated Hospital of Guangzhou Medical University, Guangzhou, China; 3 BioResource Research Center, The Third Affiliated Hospital of Guangzhou Medical University, No.63 Duobao Road, Guangzhou, Guangdong 510150 China

**Correction: BMC Pregnancy Childbirth 23**,** 508 (2023)**

**https://doi.org/10.1186/s12884-023-05784-2**

Following publication of the original article [1], the authors identified an error in Fig. 4. The correct figure is given below. The original article has been corrected.

**Fig. 4:**
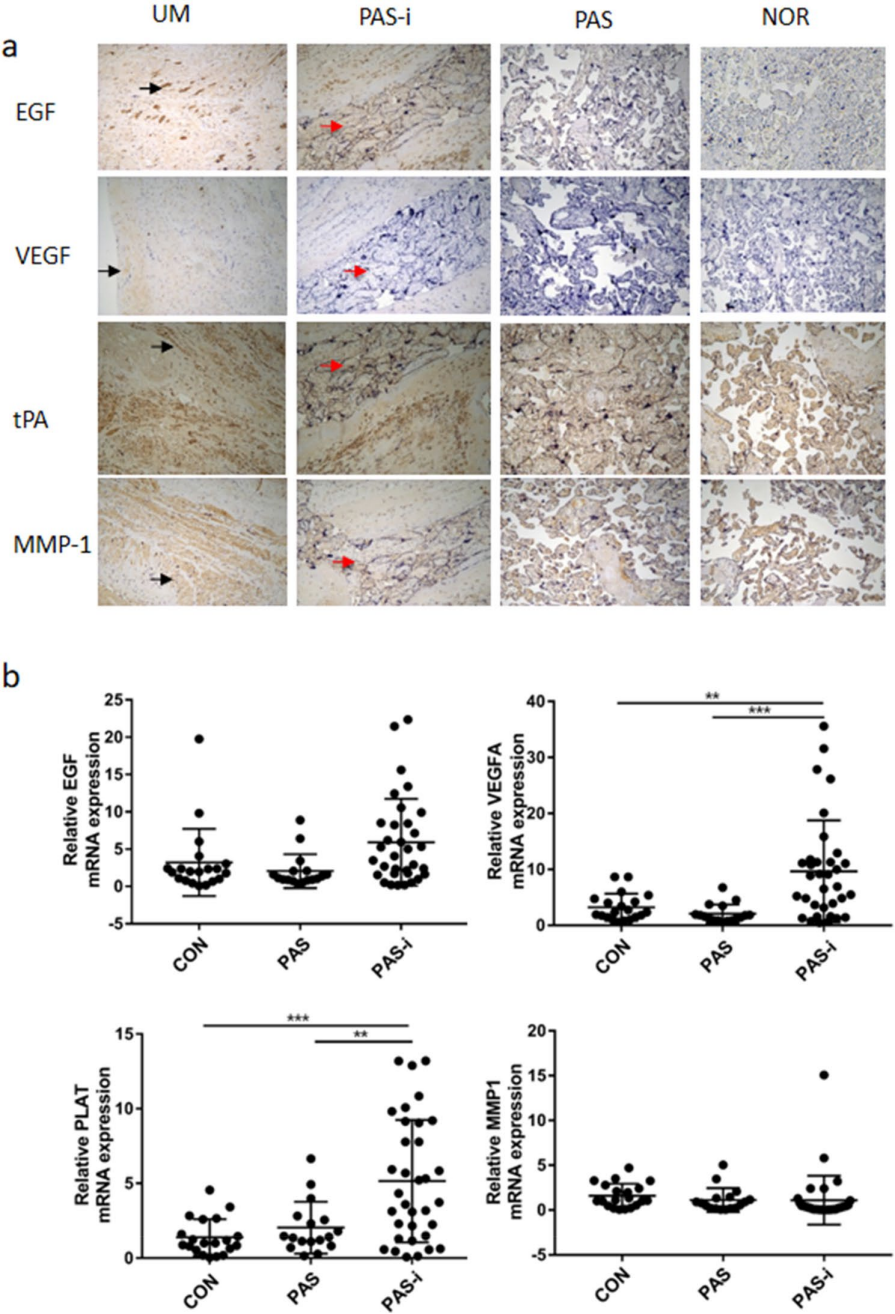
Validation of the altered biomarkers in human placenta. **a** IHC analysis of EGF, VEGF, tPA, and MMP1 in placenta from PAS cases and controls (50 × magnification; black arrows indicate uterus muscle; red arrows indicate invasive placental trophoblasts) (**b**) QPCR analysis of *EGF*, *VEGFA*, *PLAT(tPA)*, and *MMP1* in placenta from PAS cases and controls. UM: Uterus muscle; CON: placenta from normal term controls; PAS: non-invasion area of placenta from PAS patients; PAS-i: invasion area of placenta from PAS patients
