# Supplementary material for: Screening of placenta accreta spectrum disorder using maternal serum biomarkers and clinical indicators: a case–control study
Source: BMC Pregnancy Childbirth. 2023 Jul 11;23:508. doi: 10.1186/s12884-023-05784-2 (PMC10334543; doi:10.1186/s12884-023-05784-2)
Supplement: Supplementary file 1 — Additional file 1: Table S1. Serum biomarkers measured and the commercial ELISA kits used. Table S2. Primers for RT-QPCR. Table S3. Alterations in serum levels of cytokines and proteins between cases and controls in the screening group of Cohort one. Table S4. Confirmation of selected serum biomarkers for PAS screening in the validation group of Cohort one. Table S5. Specificity analysis of selected serum biomarkers for PAS screening the testing group of Cohort one. Table S6. Analysis of protein expression levels on human placenta. [file 12884_2023_5784_MOESM1_ESM.doc]

**Table S1.** Serum biomarkers measured and the commercial ELISA kits used

| Species | Protein | Company | Inter-assay CV | Sample dilution | Detection range (pg/ml) |
| --- | --- | --- | --- | --- | --- |
| Human | EGF | Abcam | ≤12% | 1:5 | 0.82-2000 |
| Human | VEGFA | Abcam | ≤4.3% | 1:1 | 15.6-1000 |
| Human | PAI1+tPA | Abcam | ≤10.2% | 1:5 | 63-4000 |
| Human | MMP1 | Abcam | ≤2.8% | 1:4 | 93.75-6000 |

**Table S2. Primers for RT-QPCR**

| Species | Gene name | Forward (5’ → 3’) | Reverse (5’ → 3’) |
| --- | --- | --- | --- |
| Human | *GAPDH* | CCACTCCTCCACCTTTGACG | AGCCAAATTCGTTGTCATACCA |
| Human | *EGF* | TGCGATGCCAAGCAGTCTGTGA | GCATAGCCCAATCTGAGAACCAC |
| Human | *MMP1* | ATGAAGTCCGGTTTTTCAAAGGG | TCCGTGTAGCACATTCTGTCC |
| Human | *VEGFA* | AGGAGGGCAGAATCATCACG | GGTCTCGATTGGATGGCAGT |
| Human | *PLAT(tPA)* | TGGTGCTACGTCTTTAAGGCGG | GCTGACCCATTCCCAAAGTAGC |

**Table S3. Alterations in serum levels of cytokines and proteins between cases and controls in the screening group of Cohort one**

| Cytokine | IL-1b | IL-2 | IL-4 | IL-6 | IL-8 | IL-10 | IL-11 | IL-16 | IL-17A |
| --- | --- | --- | --- | --- | --- | --- | --- | --- | --- |
| PAS/NOR | 1.13 | 1.98 | 1.17 | 0.86 | 1.29 | 0.92 | 0.37 | 1.04 | 1.14 |
| P value | 0.04 * | 0.26 | 0.20 | 0.84 | 0.34 | 0.36 | 0.36 | 0.72 | 0.06 |
| Cytokine | IL-17F | IL-18 | IL-21 | IL-22 | IL-23 | IL-25 | IL-28A | IL-31 | IL-33 |
| PAS/NOR | 1.05 | 1.12 | 1.11 | 1.06 | 1.16 | 1.07 | 1.00 | 1.83 | 2.62 |
| P value | 0.11 | 0.15 | 0.16 | 0.18 | 0.12 | 0.26 | 0.99 | 0.02 * | 0.09 |
| Cytokine | IL-35 | TGF-a | TGF-b1 | TGF-b2 | TGF-b3 | TIMP-1 | TIMP-3 | TIMP-4 | TNF-a |
| PAS/NOR | 1.15 | 1.05 | 1.22 | 1.09 | 1.26 | 1.19 | 1.08 | 1.06 | 1.04 |
| P value | 0.99 | 0.26 | 0.02 * | 0.00 * | 0.00 * | 0.01 * | 0.11 | 0.64 | 0.34 |
| Cytokine | MMP-1 | MMP-2 | MMP-3 | MMP-7 | MMP-8 | MMP-9 | MMP-10 | MMP-12 | MMP-13 |
| PAS/NOR | 2.01 | 0.85 | 0.81 | 0.92 | 1.42 | 1.39 | 0.99 | 1.54 | 1.01 |
| P value | 0.00* | 0.03 * | 0.05 * | 0.39 | 0.02 * | 0.01 * | 0.95 | 0.02* | 0.92 |
| Cytokine | MCP-1 | MCP-2 | MCP-3 | MCP-4 | VEGF-A | VEGF-C | VEGF-D | EGF | sCD40L |
| PAS/NOR | 0.78 | 0.95 | 1.13 | 1.05 | 1.12 | 1.13 | 0.99 | 1.44 | 2.33 |
| P value | 0.47 | 0.60 | 0.05 * | 0.73 | 0.00* | 0.00* | 0.46 | 0.00* | 0.00* |
| Cytokine | MIP-1a | MIP-3b | mip-3a | IGFBP-1 | IFN-γ | IFN-a2 | TWEAK | Haptoglobin | IP-10 |
| PAS/NOR | 1.34 | 1.12 | 1.09 | 0.69 | 1.13 | 1.19 | 1.13 | 0.87 | 1.15 |
| P value | 0.03 * | 0.60 | 0.72 | 0.01 * | 0.01* | 0.12 | 0.47 | 0.64 | 0.29 |
| Cytokine | CCL1 | CCL11 | CCL17 | CCL21 | CCL24 | CCL26 | CCL25 | CCL27 | CX3CL1 |
| PAS/NOR | 1.02 | 1.13 | 1.09 | 0.98 | 0.91 | 0.86 | 1.05 | 1.05 | 0.96 |
| P value | 0.53 | 0.01 * | 0.56 | 0.82 | 0.60 | 0.64 | 0.27 | 0.49 | 0.59 |
| Cytokine | CXCL1 | CXCL2 | CXCL5 | CXCL6 | CXCL12 | CXCL13 | CXCL16 | Osteocalcin | sCD163 |
| PAS/NOR | 1.26 | 1.37 | 1.53 | 1.54 | 1.16 | 0.62 | 1.03 | 1.02 | 0.95 |
| P value | 0.03* | 0.04 * | 0.04 * | 0.00 * | 0.06 | 0.03 * | 0.65 | 0.90 | 0.59 |
| Cytokine | MDC | MIF | MIG | MPIF-1 | sCD30 | APRIL | I-TAC | Angiopoietin-2 | STNF-R1 |
| PAS/NOR | 1.13 | 0.63 | 1.11 | 1.29 | 1.27 | 1.33 | 1.07 | 1.87 | 1.17 |
| P value | 0.24 | 0.24 | 0.37 | 0.28 | 0.01* | 0.27 | 0.56 | 0.00 * | 0.04 * |
| Cytokine | BAFF | Chitinase3 | gp130 | SIL-6Ra | PLGF | Endoglin | sFASL (35) | Pentraxin-3 | Fibrinogen |
| PAS/NOR | 0.95 | 1.19 | 0.82 | 0.95 | 2.67 | 0.78 | 1.06 | 0.80 | 1.06 |
| P value | 0.47 | 0.10 | 0.02 * | 0.45 | 0.02 * | 0.05 * | 0.10 | 0.29 | 0.36 |
| Cytokine | STNF-R2 | Hu A2M | uPA | CRP | SAP | PCT | SAA | Osteopontin | PAL-1 |
| PAS/NOR | 1.12 | 1.09 | 0.88 | 0.61 | 0.82 | 1.08 | 0.72 | 1.00 | 1.04 |
| P value | 0.06 | 0.37 | 0.10 | 0.29 | 0.00* | 0.17 | 0.29 | 1.00 | 0.52 |
| Cytokine | tPA | Ferritin | HB-EGF | TSLP |  |  |  |  |  |
| PAS/NOR | 0.81 | 1.21 | 1.00 | 0.71 |  |  |  |  |  |
| P value | 0.02 * | 0.53 | 0.89 | 0.35 |  |  |  |  |  |

Note: Data are present as the fold of PAS to NOR groups. PAS, pregnant women with placenta accreta spectrum disorders. NOR, normal term controls. Statistical *p* values are calculated by unpaired *t*-test. *: compared to NOR group by unpaired *t*-test, *P* < 0.05.

**Table S4. Confirmation of selected serum biomarkers for PAS screening in the validation group of Cohort one**

| Cytokine | IL-17A | IL-17F | IL-19 | IL-22 | IGFBP-1 | tPA |
| --- | --- | --- | --- | --- | --- | --- |
| PAS/NOR | 0.96 | 0.73 | 0.97 | 0.86 | 0.94 | 0.69 |
| P value | 0.20 | 0.15 | 0.08 | 0. 18 | 0.71 | 0.01* |
| Cytokine | CD30 | Endoglin | sCD40L | Angiopoietin-2 | Fas Ligand | TIMP-1 |
| PAS/NOR | 1.13 | 0.87 | 1.22 | 0.82 | 0.93 | 1.08 |
| P value | 0.04* | 0.18 | 0.38 | 0.39 | 0.39 | 0.31 |
| Cytokine | CXCL2/GRO β | CCL15/MIP-1δ | CXCL6/GCP-2 | gp130 | SAP | MCP-3 |
| PAS/NOR | 0.80 | 0.80 | 1.00 | 1.04 | 0.76 | 1.00 |
| P value | 0.14 | 0.18 | 0.97 | 0.42 | 0.22 | 0.95 |
| Cytokine | MMP-1 | MMP-2 | MMP-3 | MMP-8 | MMP-9 | MIP-1α |
| PAS/NOR | 1.77 | 0.06 | 0.87 | 1.54 | 1.39 | 0.98 |
| P value | 0.03 * | 0.31 | 0.10 | 0.02 * | 0.02 * | 0.95 |
| Cytokine | VEGF-A | VEGF-C | EGF | TGF-B1 | TGF-B2 | TGF-B3 |
| PAS/NOR | 3.87 | 0.86 | 1.56 | 0.98 | 1.00 | 1.01 |
| P value | 0.01* | 0.44 | 0.01 * | 0.74 | 0.95 | 0.46 |
| Cytokine | BLC | Eotaxin | PIGF-1 |  |  |  |
| PAS/NOR | 0.76 | 1.08 | 0.94 |  |  |  |
| P value | 0.10 | 0.45 | 0.29 |  |  |  |

Note: Data are present as the fold of PAS to NOR groups. PAS, pregnant women with placenta accreta spectrum disorders. NOR, normal term controls. *: compared to NOR group by unpaired *t*-test, *P* < 0.05.

**Table S5. Specificity analysis of selected serum biomarkers for PAS screening the testing group of Cohort one**

|  | EGF | VEGF-A | tPA | MMP-1 |
| --- | --- | --- | --- | --- |
| PAS/PP | 1.70 | 8.61 | 1.02 | 3.35 |
| P value | 0.04* | 0.02* | 0.86 | 0.04* |
| PAS/PE | 2.82 | 4.52 | 0.42 | 2.56 |
| P value | 0.00* | 0.05* | 0.00* | 0.09^#^ |

Note: Data are present as the fold of PAS to P or PE groups. PAS, pregnant women with placenta accreta spectrum. PE, pregnant women with preeclampsia. PP, pregnant women with placenta previa.

Statistical *p* values are calculated by unpaired *t*-test.

^#^: *P* < 0.05 and *: *P* < 0.05, compared between two groups by unpaired *t*-test.

**Table S6.** **Analysis of protein expression levels on human placenta**

| Cytokine | Sample | D0 | D1 | D2 | D3 | Ave | P value |
| --- | --- | --- | --- | --- | --- | --- | --- |
| EGF | NOR | 0 | 31 | 48 | 22 | 1.91 | 0.000* |
|  | PAS | 0 | 6 | 14 | 33 | 2.51 |  |
|  | PAS-i | 0 | 9 | 4 | 8 | 1.95 |  |
| VEGF | NOR | 95 | 5 | 0 | 0 | 0.05 | 0.002* |
|  | PAS | 51 | 4 | 0 | 0 | 0.08 |  |
|  | PAS-i | 15 | 6 | 0 | 0 | 0.29 |  |
| MMP-1 | NOR | 0 | 42 | 34 | 24 | 1.82 | 0.000* |
|  | PAS | 2 | 10 | 20 | 21 | 2.13 |  |
|  | PAS-i | 6 | 3 | 4 | 8 | 1.67 |  |
| tPA | NOR | 3 | 30 | 34 | 34 | 1.98 | 0.000* |
|  | PAS | 0 | 10 | 19 | 24 | 2.26 |  |
|  | PAS-i | 6 | 4 | 3 | 8 | 1.62 |  |

Note: NOR: placenta from normal term controls; PAS: non-invasion area of placenta from PAS patients; PAS-i: invasion area of placenta from PAS patients.

D0: Absent staining; D1: Weak staining; D2: Moderate staining;D3: Strong staining.

*: *P* < 0.05 using *X*² test among the 3 groups.
